# Composite likelihood estimation of demographic parameters

**DOI:** 10.1186/1471-2156-10-72

**Published:** 2009-11-12

**Authors:** Daniel Garrigan

**Affiliations:** 1 Department of Biology, University of Rochester, Rochester, New York, USA

## Abstract

**Background:**

Most existing likelihood-based methods for fitting historical demographic models to DNA sequence polymorphism data to do not scale feasibly up to the level of whole-genome data sets. Computational economies can be achieved by incorporating two forms of pseudo-likelihood: composite and approximate likelihood methods. Composite likelihood enables scaling up to large data sets because it takes the product of marginal likelihoods as an estimator of the likelihood of the complete data set. This approach is especially useful when a large number of genomic regions constitutes the data set. Additionally, approximate likelihood methods can reduce the dimensionality of the data by summarizing the information in the original data by either a sufficient statistic, or a set of statistics. Both composite and approximate likelihood methods hold promise for analyzing large data sets or for use in situations where the underlying demographic model is complex and has many parameters. This paper considers a simple demographic model of allopatric divergence between two populations, in which one of the population is hypothesized to have experienced a founder event, or population bottleneck. A large resequencing data set from human populations is summarized by the joint frequency spectrum, which is a matrix of the genomic frequency spectrum of derived base frequencies in two populations. A Bayesian Metropolis-coupled Markov chain Monte Carlo (MCMCMC) method for parameter estimation is developed that uses both composite and likelihood methods and is applied to the three different pairwise combinations of the human population resequence data. The accuracy of the method is also tested on data sets sampled from a simulated population model with known parameters.

**Results:**

The Bayesian MCMCMC method also estimates the ratio of effective population size for the X chromosome versus that of the autosomes. The method is shown to estimate, with reasonable accuracy, demographic parameters from three simulated data sets that vary in the magnitude of a founder event and a skew in the effective population size of the X chromosome relative to the autosomes. The behavior of the Markov chain is also examined and shown to convergence to its stationary distribution, while also showing high levels of parameter mixing. The analysis of three pairwise comparisons of sub-Saharan African human populations with non-African human populations do not provide unequivocal support for a strong non-African founder event from these nuclear data. The estimates do however suggest a skew in the ratio of X chromosome to autosome effective population size that is greater than one. However in all three cases, the 95% highest posterior density interval for this ratio does include three-fourths, the value expected under an equal breeding sex ratio.

**Conclusion:**

The implementation of composite and approximate likelihood methods in a framework that includes MCMCMC demographic parameter estimation shows great promise for being flexible and computationally efficient enough to scale up to the level of whole-genome polymorphism and divergence analysis. Further work must be done to characterize the effects of the assumption of linkage equilibrium among genomic regions that is crucial to the validity of applying the composite likelihood method.

## Background

The availability of whole-genome polymorphism data offers both great opportunities and tremendous challenges to the study of population genetics. Complete genotype information from populations allows increased resolution of parameters in complex evolutionary or demographic models. The challenge is to develop computational methods that permit the efficient use of such large-scale datasets. Likelihood-based coalescent methods have proven very flexible for the analysis of DNA sequence polymorphism. However full likelihood methods, such as Markov chain Monte Carlo (MCMC) and Importance Sampling (IS), are not efficient enough to scale up to genome-wide datasets, necessitating the use of approximate methods for estimating likelihoods. One problem with existing MCMC and IS methods is that a proposal function must be employed to efficiently search among candidate coalescent histories. To circumvent this problem, approximate likelihood methods have proven useful. This class of methods reduces the dimensionality of a full DNA polymorphism dataset to a set of summary statistics, thereby also reducing the number of coalescent histories that need to be sampled to obtain an estimate of the likelihood.

One potential drawback of approximate likelihood methods is that a significant amount of information contained in the original data may be lost. A second problem with full MCMC and IS methods is that integrating over the entire space of possible histories for partially linked polymorphisms along a chromosome can quickly become computationally intractable. In this regard, composite likelihood has been shown to be a promising method for the analysis of partially linked polymorphisms [1-3]. Using this method, the likelihood function is computed marginally for each polymorphism (or contiguous sets of linked polymorphisms) and their product is taken to be an approximation of the full likelihood [4]. Because composite likelihood methods are found to yield consistent estimators of population parameters when the number of regions examined becomes very large [5,6], they may be particularly applicable to whole-genome datasets.

One of the commonly used class of models in population genetics aims to quantify divergence time by measuring the genetic distance between two populations or species. Yet many measures of genetic distance are susceptible to biases introduced by non-equilibrium conditions during the histories of the populations. Specifically, evolutionary forces that reduce within-population variation are known to inflate measures of genetic distance; such forces may include natural selection [7] or temporal fluctuations in the effective population size [8-10]. In contrast to the locus-specific effects of natural selection, fluctuations in effective size, such as population bottlenecks, are expected to influence the frequencies of alleles throughout the entire genome and therefore should be readily detectable using genome-wide polymorphism data. Thus, it is desirable to develop methods that can not only estimate divergence time from genome-wide polymorphism data, but can also simultaneously account for non-equilibrium demographic events, such as population bottlenecks.

One novel implementation of a coalescent-based method that simultaneously estimates divergence time between two populations and accounts for population bottlenecks is described by Li and Stephan [11]. This method achieves the necessary computational economies by summarizing two-population polymorphism data in the form of the joint frequency spectrum. The joint frequency spectrum is a two-dimensional matrix whose elements are the frequencies of the derived nucleotide allele in a joint sample from two populations or species. Using the joint frequency spectrum, mutations can be classified as either fixed, shared, or exclusive to one of the populations [12]. Li and Stephan [11] estimate divergence time and bottleneck parameters from a joint frequency spectrum constructed from 250 X-linked loci, representing samples of African and non-African populations of *Drosophila melanogaster*.

**Figure 1:**
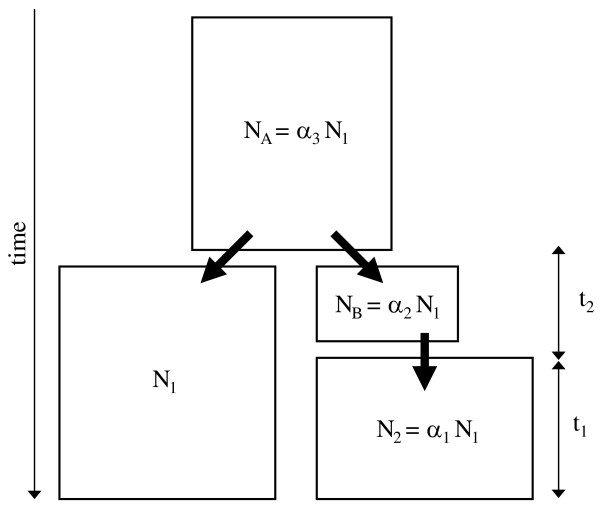
**Demographic model**. A schematic of the two population divergence model that is fit to the joint frequency spectrum. Looking forward in time, the ancestral population splits *t*_1 _+ *t*_2 _generations before the present into two descendant populations. At this time, the effective size of population 1 is assumed to be *N*_1 _and the founding size of population 2 is assumed to be *α*_2_*N*_1_. Then, after *t*_2 _generations, population 2 grows to effective size *α*_1_*N*_1_. Lastly, the effective size of the common ancestral population is assumed to be *α*_3_*N*_1_. Thus, the divergence model is governed by five parameters that need to estimated: *t*_1_, *t*_2_, *α*_1_, *α*_2 _and *α*_3_.

While the approach of Li and Stephan [11] does provide an economical method for fitting a parameter-rich population divergence model to a large polymorphism dataset, it can nonetheless be further economized and extended. Because the authors consider linkage disequilibrium among polymorphisms within loci, the mutation rate per locus must be included as a parameter. In contrast, by adopting a composite likelihood approach and assuming linkage equilibrium among polymorphism loci, it is possible to eliminate the mutation rate as a parameter, similar to a recent approach by Hernandez et al. [13]. Lastly, Li and Stephan [11] used a maximum likelihood method that evaluates a fixed set of proposed parameter values for their model. This approach does not capitalize on advances in Bayesian MCMC methods for model parameter estimation. The present study extends the approach of Li and Stephan [11] by both eliminating the mutation rate as a nuisance parameter and implementing a Bayesian MCMC approach that takes advantage of multiprocessor/multicore computer architecture. The proposed method is tested for accuracy using simulated joint frequency spectra and is then applied to three large autosomal and X-linked resequence datasets from African, European, Asian, and Oceanian human populations [14]. Three pairwise analyses of the populations are performed to estimate the parameters of an "Out-of-Africa" bottleneck model (Figure 1), paying particular attention to the effective population size of the X chromosome *versus *the autosomes.

## Results and Discussion

### Behavior of the Markov Chain

To gain confidence regarding the convergence of Markov chains to their stationary distribution, it is important that the chains mix well and also that independent runs of the chains converge to the same posterior probability distribution. The mixing of independently seeded chains is assessed by measuring the autocorrelation of parameter values accepted from the prior probability distributions. Autocorrelations are measured at lag intervals from 1 to 50. Table 1 presents the autocorrelations, at lag 50 (*ρ*_50_) for each parameter value, over all ten replicate runs. For each of the six different datasets shown in the table, the two time parameters show the weakest mixing behavior. Similarly, for each dataset, the two time parameters showed the highest levels of cross-correlation, ranging between -0.2 and -0.4 (data not shown). Interestingly, the parameter that shows the best mixing behavior is the ancestral population size scaling factor *α*_3_. The potential scale reduction factor (PRSF) and the upper 97.5% quantile of the PRSF distribution are all very close to unity (Table 1) for every dataset, except simulated data set G, which differs from the others in that much longer divergence times are involved. A PRSF value significantly greater than one implies that chains must be run longer to achieve convergence to the stationary distribution.

**Table 1:** Markov chain statistics.

Dataset	Statistic	*t*_1_	*t*_2_	*h*	*α*_1_	*α*_2_	*α*_3_
AA	*ρ*_50_	0.1520	0.1478	0.0353	0.0600	0.0530	0.0099
	PSRF	1	1	1	1	1	1
	Upper PSRF	1	1	1	1	1	1
AE	*ρ*_50_	0.1587	0.1382	0.0302	0.0565	0.0349	0.0044
	PSRF	1	1	1	1	1	1
	Upper PSRF	1	1	1	1	1	1
AO	*ρ*_50_	0.1617	0.1324	0.0133	0.0497	0.0326	0.0081
	PSRF	1	1	1	1	1	1
	Upper PSRF	1	1	1	1	1	1
A	*ρ*_50_	0.1197	0.2637	0.0868	0.0269	0.0538	0.0669
	PSRF	1	1.02	1	1.02	1	1
	Upper PSRF	1.01	1.03	1	1.02	1	1
D	*ρ*_50_	0.1217	0.1916	0.0728	0.0232	0.0604	0.0570
	PSRF	1	1.01	1	1.01	1	1
	Upper PSRF	1	1.02	1	1.01	1	1
G	*ρ*_50_	0.1066	0.1567	0.1097	0.0938	0.0053	0.0805
	PSRF	1.13	1.19	1.11	1	1.15	1.27
	Upper PSRF	1.27	1.39	1.22	1	1.30	1.53

The behavior of the Markov chains for all datasets, as determined by ten independent runs of the chain for each dataset. Three statistics are given: *ρ*_50 _is the autocorrelation of each parameter at lag 50 steps, PSRF refers to the potential scale reduction factor for each parameter, and Upper PRSF is the 97.5% quantile of the PRSF. Multivariate PRSF values are not given. The resequencing data sets of Wall et al. [14] are abbreviated as Africa-Asia (AA), Africa-Europe (AE), and Africa-Oceania (AO). Additionally, autocorrelation values are also presented for three representative simulated data sets (A, D, and G).

### Simulated Datasets

The performance of the method under a true population divergence model is assessed using twelve simulated joint frequency spectra. Table 2 lists the parameter values for each of the twelve simulated data sets presented here. The simulated data sets are intended to represent both recent population bottlenecks (A-F), as well as older population bottlenecks (G-L). The duration of the reduction phase of the bottleneck is the same in all of the simulated data sets, however, populations either experience a ten-fold reduction or no reduction at all, in which case the model reduces to one of pure population growth (C, F, I, L). Similarly, during the recovery phase of the bottlenecks, populations can grow by either 100-fold or 1000-fold. Lastly, the ratio of the X chromosome effective population size to that of the autosomes varies between three-quarters (expected if there are an equal number of breeding males and females) and unity (a 7:1 ratio of reproducing females to males).

**Table 2:** Parameters for simulated data.

Dataset	*t*_1_	*t*_2_	*h*	*α*_1_	*α*_2_	*α*_3_
A	0.05	0.03	0.75	10	0.1	1
B	0.05	0.03	0.75	100	0.1	1
C	0.05	0.03	0.75	100	1	1
D	0.05	0.03	1	10	0.1	1
E	0.05	0.03	1	100	0.1	1
F	0.05	0.03	1	100	1	1
G	1	0.03	0.75	10	0.1	1
H	1	0.03	0.75	100	0.1	1
I	1	0.03	0.75	100	1	1
J	1	0.03	1	10	0.1	1
K	1	0.03	1	100	0.1	1
L	1	0.03	1	100	1	1

The population bottleneck model parameters used to generate simulated datasets to test the accuracy of the proposed composite likelihood method. Parameters *t*_1 _is the time since the bottleneck recovery, *t*_2 _is the duration of the bottleneck, *h *is the ratio of the effective population size of the X compared to the autosomes, *α*_1 _is the current size of the bottlenecked population relative to the non-bottlenecked population, *α*_2 _is the relative size of the bottlenecked population during the reduction phase, and *α*_3 _is the relative size of the ancestral population. Both time parameters are in units of twice the current effective population size of the non-bottlenecked population.

**Figure 2:**
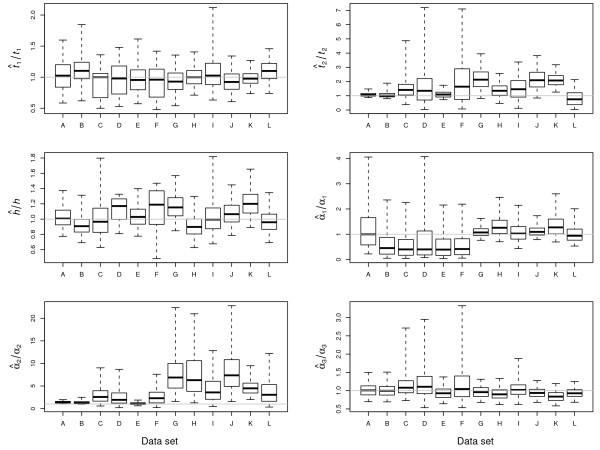
**Simulated data estimates**. Accuracy of parameter estimation for the twelve parameters in the divergence model. For each parameter, the ratio of the estimated median posterior probability to the "true" value of the parameter in the simulation. The horizontal gray lines delineate a ratio of unity. The heavy lines in the box plots are the median, the hinge of the boxes are the 25% and 75% quantiles and the outer whiskers represent the 2.5% and 97.5% quantiles. Results are presented for each of the twelve simulated datasets, the parameters of which are listed in Table 1. Posterior probability distributions are taken over all ten replicate runs of the Markov chain.

The posterior probability distributions shown in Figure 2 illustrate several consistencies, as well as several systematic biases in the MCMCMC estimation procedure. For both recent and ancient population bottlenecks, the time of recovery (*t*_1_) is estimated accurately. However, the duration of the bottleneck (*t*_2_) tends to be slightly, and consistently, overestimated when the bottleneck occurred 2*N*_1 _generations ago (data sets G-L). When the bottleneck is recent, the MCMC method tends to systematically underestimate the current effective population size of population 2 (*α*_1_), regardless of whether it is 100 or 1000 times that of the founding population size. However, underestimation does not appear to be a problem for the data sets obtained from simulations of an older bottleneck time. Also, the size of the founder population (*α*_2_) tends to be consistently overestimated when the bottleneck is ancient. In all cases, the ancestral population size (*α*_3_) is estimated accurately, which is compatible with the results of Becquet and Przeworski [15]. Lastly, the ratio of the effective population size of the X chromosome to that of the autosomes (*h*) is estimated accurately, however, in most cases, the 95% HPD interval includes the value of the parameter expected under an alternate case of interest. For example, Figure 2 shows that the median values of  tend to slightly overestimate the true value of *h *= 1, and that, in all but four cases (D, E, J, and K), the 95% HPD interval includes 0.75 when the true *h *value is unity and also when the true value of *h *is three-fourths the 95% HPD interval includes unity. This observation suggests that the MCMCMC method may not always have the adequate power to reject the null hypothesis that *h *= 3/4. The effect that analyzing a larger data set may have on this power remains to be investigated.

### Estimates of Human Bottleneck Parameters

**Figure 3:**
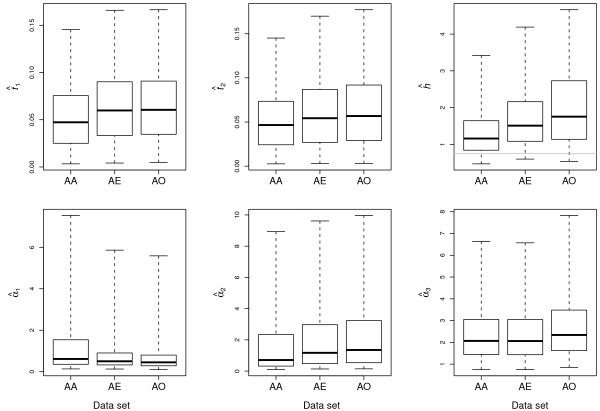
**Human data estimates**. Representations of the posterior probability distributions for the six divergence model parameters from the data of Wall et al. [14]. Three pairwise population comparisons are plotted: Africa-Asia (AA), Africa-Europe (AE), and Africa-Oceania (AO). The heavy lines in the box plots are the median, the hinge of the boxes are the 25% and 75% quantiles and the outer whiskers represent the 2.5% and 97.5% quantiles. Numerical values for the median and 95% highest posterior density intervals can be found in Table 3. In the plot of the ratio of the X chromosome to autosomal effective population size (*h*), the horizontal gray line delineates a ratio of 3/4. As in Figure 2, the posterior probability distributions shown here are taken over all ten replicate runs of the Markov chain.

**Figure 4:**
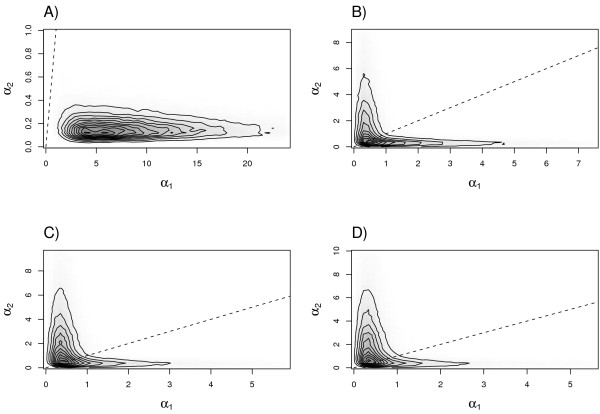
**Evidence for population growth**. Joint posterior density plots for the *α*_1 _and *α*_2 _parameters for four different data sets: A) simulated data set A, B) Africa-Asia, C) Africa-Europe, and D) Africa-Oceania. The dashed line plots the case of *α*_1 _= *α*_2_, which is indicative of no recent population growth. In panel A, the posterior density for the simulated bottleneck data lies below the dashed line, supporting recent population growth. However, in the other three panels representing the empirical resequence data of [14], the joint posterior density lies within the one-to-one region, suggesting a lack of evidence for recent population growth.

The quantiles of the marginal posterior probability distributions obtained by applying the method to the resequence data of Wall et al. [14] are shown in Figure 3, with each of the three comparisons between continental human populations shown side by side. Also, Table 3 provides the numerical values for the median estimated parameter values and the corresponding 95% HPD intervals. It should be first noted that none of these results are consistent with a population bottleneck model. In each of the three comparisons, the ancestral effective population size is estimated to be twice that of the current Mandenka effective population size and the median estimated values of neither *α*_1 _or *α*_2 _are greater than one. Figure 4 plots the joint posterior probability distributions of *α*_1 _and *α*_2 _for simulated bottleneck data set A and the three empirical resequencing data sets. These joint distributions confirm that the method accurately detects recent population growth from data simulated under a bottleneck model, but is also unable to support recent population growth for the data of Wall et al. [14]. The resequence data are consistent with a model of a reduced effective population size with no subsequent expansion for any of the four sampled human populations.

**Table 3:** Estimates of human demographic parameters.

Dataset	*t*_1_	*t*_2_	*h*	*α*_1_	*α*_2_	*α*_3_
AA	0.0473	0.0466	1.1582	0.6148	0.7045	2.0666
	(0.0033-0.1454)	(0.0026-0.1451)	(0.4642-3.4620)	(0.1367-7.5359)	(0.0933-8.9268)	(0.7494-6.6319)
AE	0.0598	0.0543	1.5091	0.5035	1.1674	2.0612
	(0.0042-0.1659)	(0.0029-0.1699)	(0.5987-4.1903)	(0.1286-5.8619)	(0.1358-9.6137)	(0.7549-6.5703)
AO	0.0605	0.0568	1.7542	0.4511	1.3512	2.3385
	(0.0049-0.1665)	(0.0031-0.1772)	(0.5381-4.6644)	(0.1016-5.5873)	(0.1424-9.9650)	(0.8543-7.8275)

The posterior probability medians of the five parameters in the demographic model. The 95% highest posterior density interval for each parameter is given in the line below the median values. Values are listed for each of three data sets, including the Africa-Asia comparison (AA), the Africa-Europe (AE) comparison, and the Africa-Oceania comparison (AO).

The divergence time of African and non-African populations (*t*_*d*_) is consistent across comparisons. The estimated median Africa-Asia divergence time is 0.1010 × 2*N*_1 _with a 95% HPD interval of 0.0416-0.2077. The estimated median Africa-Europe divergence time is 0.1209 × 2*N*_1 _with a 95% HPD interval of 0.0524-0.2422. Lastly, the estimated median of the Africa-Oceania divergence time is 0.1254 × 2*N*_1 _with a 95% HPD interval of 0.0536-0.2458. If the current effective population size of the Mandenka is assumed to be on the order of 10^4 ^and that the human generation time is 25 years, these numbers correspond to 50,500 years for the Africa-Asia divergence, 60,450 years for the Africa-Europe divergence, and 62,700 years for the Africa-Oceania divergence. There is no support for the hypothesis that these estimated times significantly differ from one another and therefore, a single Africa/non-Africa divergence event cannot be rejected.

There is some suggestion that the rate of coalescence, after divergence from the African population, may be higher in the Asian population than in the other two non-African populations. This can be seen by examining the intensity of the effective population size reduction phase (*F *= *t*_2_/*α*_2_). For the Africa-Asia comparison the estimated median *F *is 0.1027 with a 95% HPD interval of 0.0010-0.2297, while the estimated median *F *for the Africa-Europe comparison is 0.0603 (95% HPD interval of 0.0019-0.2035) and 0.05122 for the Africa-Oceania comparison (95% HPD interval of 0.0012-0.1936). While the estimated median *F *from the Africa-Asia comparison does not lie outside the 95% HPD intervals of the other two comparisons, the difference is much more pronounced than the difference between divergence time estimates, yet cannot be considered conclusive evidence. Lastly, the estimated median ratios of effective population sizes for the X chromosome to that of the autosomes is greater than 3/4 for all comparisons. The model assumes a single value of *h *for all populations in the model. The highest estimated median *h *is found in the Africa-Oceania comparison, while the smallest is found in the Africa-Asia comparison (Table 3).

## Conclusion

Efficient computational methods for fitting complex demographic or evolutionary models to large genomic datasets present a great challenge to population geneticists. The method presented here uses two approximations to achieve the necessary computational efficiencies. The first is an approximate likelihood method, in which large genomic polymorphism datasets are summarized in terms of the joint frequency spectrum. This approach reduces the number of coalescent genealogies that must be sampled to obtain an estimation of the likelihood, compared with most full likelihood-based approaches. Secondly, this methodology is rendered feasible by a composite likelihood approach, which assumes that all polymorphic sites are in linkage equilibrium and have independent genealogical histories. The method is implemented using a model of allopatric population divergence, with a founder event occurring in the history of one of the two diverging populations.

Simulated datasets are used to investigate the accuracy of the MCMC parameter estimation. The method is found to perform well, although it experiences some difficulty delineating the time of the founding event versus the time of population growth parameters, even though the intensity of the bottleneck is estimated accurately. The problem of bottleneck models being partially identifiable, with respect to the timing and magnitude of the reduction phase, was also observed by several other investigators [16-18]. This suggests that current approaches to estimating bottleneck parameters may be limited to estimating the total amount of drift occurring during the reduction phase of the bottleneck (e.g., the product of the bottleneck duration and the magnitude). Again, it remains to be determined what effect the size of a data set may have on this problem of identifiability.

The composite likelihood method is applied to three joint frequency spectra datasets constructed from samples of four continental human populations [14]. The results indicate that there is evidence for a reduction in both the African and non-African effective population sizes, but no evidence that this reduction was followed by a recovery in size that is characteristic of population bottlenecks. As noted by Fay and Wu [19], there is expected to be a period of time, following a population bottleneck, during which the X and the autosomes lag in their signal of population growth compared to the Y chromosome and mitochondrial DNA (i.e., a slower accumulation of rare mutations than the two haploid compartments of the genome).

The conclusion gleaned from the present analysis stands in contrast to those of Voight et al. [17], which is the only other resequencing study to test a population bottleneck model explicitly. Voight et al. use a variant on approximate likelihood to infer bottleneck parameters from 50 resequenced autosomal loci; their analysis was performed separately for datasets constructed from an African, European, and Asian sample. Voight et al. conclude that their African sample cannot reject a constant-size population model, while a bottleneck model is supported by the two samples of non-African populations. While it is clear that the analysis presented by Voight et al. supports a reduction in the effective population size of non-African populations, their evidence for a recovery period (growth) from autosomal data appears to depend upon a set of assumptions, including the absolute value of parameters such as the size of the ancestral population and the severity of the bottleneck, for which reliable estimates do not yet exist. Thus, it appears far from conclusive that there is convincing evidence for recent population growth from either autosomal or X-linked non-coding resequence datasets.

The differential recovery time for the X chromosome versus the autosomes has also prompted Pool and Nielsen [20] to suggest that the X chromosome may recover rare variants more quickly than the autosomes following a population bottleneck and that this could elevate the ratio of X-linked to autosomal nucleotide diversity. Indeed, the human data analyzed here show equivalent levels of X-linked and autosomal diversity [21]. The composite likelihood analysis suggests that, for this dataset, the effective population size of the X chromosome is equal to, or greater than, that of the autosomes, even after taking into consideration that effects of a bottleneck. The effect described by Pool and Nielsen [20] would elevate the X to autosomal diversity ratio upwards of 1,000 generations following the recovery period of the bottleneck.

The conclusion presented here, that a bottleneck alone is insufficient to produce the observed elevation in X-linked diversity, indirectly supports the conclusions of Hammer et al. [21], that a systematic skew in the breeding sex-ratio is responsible for the X to autosome diversity ratio. However, if the expected ratio of X chromosome effective population size to that of the autosomes, under a purely neutral demographic model, is *h *= 9/(8(2 - *ϕ*)), where *ϕ *is the proportion of the population that is female, then lim_*ϕ*→1 _*h *= 9/8. This expected maximum value for the ratio of X to autosomal effective population size is lower than all three of our median estimates of *h*. Although the lower bound of the 95% HPD interval for *h *varies between 0.46 and 0.60 for the three comparisons, the median estimates suggest that there may be additional forces, such as sex-biased migration, that act at a genome-wide scale. Interestingly, the conclusions reached here contrast with those of Keinan et al. [22], who use single nucleotide polymorphism (SNP) genotype data to infer a lower effective population size for the X chromosome. These contrasting conclusions may reflect the different types of data used in the analyses (resequence versus SNP-typing) or the influence of natural selection near regions of the genome with a high density of coding sequence. Undoubtedly, this dichotomy will be resolved by the forthcoming data from the 1,000 Genomes Project.

The combination of approximate and composite likelihood methods is a promising approach for scaling up population genetics analyses to the level of whole-genome polymorphism data, yet much remains to be done to characterize the validity and accuracy of these methods. Projects are underway to examine further the properties of this method and to apply it to a full-genome polymorphism dataset.

## Methods

### Coalescent Model and Likelihood Estimation

The proposed composite/approximate likelihood method is applied to a model of allopatric population divergence, in which one of the populations experiences a transient founder event. This scenario is modeled by the coalescent process. Looking backwards in time, two populations with effective sizes *N*_1 _and *N*_2 _= *α*_1_*N*_1_. The second population changes its effective population size at time *t*_1 _to *N*_*B *_= *α*_2_*N*_1_. Then at time *t*_1 _+ *t*_2_, the two populations are descended from an ancestral population of size *N*_*A *_= *α*_3_*N*_1 _(Figure 1). Therefore, the divergence time of the two populations is *t*_*d *_= *t*_1 _+ *t*_2 _and time is measured in units of 2*N*_1 _generations before the present. Thus, the model consists of four different rates of coalescence and its behavior is governed by a total of six parameters, which are collectively referred to as the vector ***λ ***= {*t*_1_, *t*_2_, *α*_1_, *α*_2_, *α*_3_}.

The coalescent process underlying this model traces the ancestry of *n*_1 _and *n*_2 _chromosomes sampled from each of the two populations, respectively. The total number of chromosomes in the joint sample is *n *= *n*_1 _+ *n*_2_. The number of ancestral lineages remaining within each of the two populations decays independently as time is traced backwards, such that there will be 1 ≤ *k*_1 _≤ *n*_1 _sampled lineages remaining in population 1 and 1 ≤ *k*_2 _≤ *n*_2 _lineages remaining in population 2. At time *t*_*d*_, the remaining sampled lineages merge so that *k *= *k*_1 _+ *k*_2_, at which point they are exchangeable and continue to coalesce until the most recent common ancestor of the joint sample. For *t *<*t*_*d*_, the rate of coalescence for the joint sample is the sum of two independent exponential distributed rates. The total coalescence rate (*u*) is given by(1)

Given that a coalescent event occurs and *t *<*t*_*d*_, the probability that two randomly chosen lineages in population 1 coalesce is(2)

The probability that the coalescent event occurs in population 2 is simply Pr (*c*_2_) = 1 - Pr(*c*_1_). When a separate joint frequency spectrum representing X-linked data is also being considered, then the parameter *h *scales both *t*_1 _and *t*_2_, such that *t*_1_(X) = *t*_1_(A)/*h *and *t*_2_(X) = *t*_2_(A)/*h*, where t_* _(X) is the time of the event for X-linked loci and t_* _(A) is the time of the event for autosomal loci. Under the assumption of neutral evolution, when the male and females population sizes are equal, *h *is expected to be 3/4.

A bifurcating coalescent genealogy consists of 2*n *- 2 branches. Each branch in the genealogy can be labeled *b*_*z *_for 1 ≤ *z *≤ 2*n *- 2. By drawing from an exponential distribution given by equation (1), each branch can be assigned a length *T*_*z*_. Let the total length of the entire genealogy be the summation . Now let *B*_*ij *_be the set of all branches in a given genealogy that have *i *descendants in a sample of *n*_1 _chromosomes from population 1 and *j *descendants in a sample of *n*_2 _chromosomes from population 2. The sum of the lengths of all branches in the set *B*_*ij *_for a given genealogy is(3)

where *I *(*b*_*z *_∈ *B*_*ij*_) is a boolean variable indicating membership of branch *b*_*z *_in the set *B*_*ij*_.

Furthermore, assume an infinite sites model in which *μ *is the rate of mutation for a given variable nucleotide position and that this rate is diminishingly small (e.g., *μ *→ 0). Conditioned on that nucleotide site being polymorphic and the genealogy for that site (), the probability of sampling a mutant nucleotide with frequency *i *in population 1 and *j *in population 2 is(4)

[23]. These assumptions eliminate the mutation rate as a nuisance parameter in the model. If a total of *r *independent Monte Carlo samples are generated, the probability of sampling a polymorphism with configuration (*i*, *j*) can be approximated(5)

where Pr (_*r*_|***λ***) is the probability of the *r*th coalescent genealogy. To avoid the zero-frequency problem, in cases where Pr (*i*, *j*|***λ***) = 0, Laplace's rule of succession is applied and a negligible probability density equal to(6)

is added to the zero probability entry. Although this pseudocount is somewhat arbitrary, it is equal to a single random branch length drawn from the entire Monte Carlo sample of size *r*. Finally, the likelihood of ***λ***, given the entire observed joint frequency spectrum (**S**) from a total of ℒ polymorphic nucleotide sites, can be approximated by(7)

where **S**_*ij *_is the number of sites with configuration (*i*, *j*) and provided that *i *+ *j *is not equal to zero or *n*_1 _+ *n*_2_, since neither of these two conditions would result in a polymorphism at that site. A total of *r *= 10^5 ^coalescent genealogies are sampled to estimate Pr(*i*, *j*|***λ***).

By taking the product of the marginal likelihoods across all ℒ polymorphic nucleotide sites in equation (7), it is assumed that all sites are in linkage equilibrium and, therefore, have independent coalescent genealogies. This means that equation (7) belongs to a class of approximate methods known as composite likelihood [4]. There is some suggestion that composite likelihood estimators of population parameters are consistent, particularly when the number of regions examined becomes very large. While the data of Wall et al. [14] consist of only 40 independent regions of the genome, the method may be promising for future analyses of whole-genome polymorphism data. One potential consequence of linkage disequilibrium among sites may be that the resulting credible intervals for the MCMC parameter estimation may be too narrow.

### Metropolis-coupled Markov chain Monte Carlo (MCMCMC) Parameter Inference

A Bayesian approach is used to estimate the model parameters in ***λ ***from the observed joint frequency spectrum data (**S**). The parameters in ***λ ***constitute the state of a Markov chain that relies upon equation (7) to sample from its stationary distribution,(8)

where *f*(***λ***) is the prior density of parameter values and *f*(**S**) = ∫*L*(**S**|***λ***) *f*(***λ***)*d****λ ***is a normalizing constant. The prior densities employed in this study were chosen from repeated exploratory runs of the Markov chain. For the analysis of the human resequence data, the prior distributions for the two time parameters are uniform over the interval (0, 1), while the prior for the ratio of X chromosome to autosomal effective population size is uniform over the interval (0, 5). The prior densities for the relative effective population size parameters are exponential with mean 3. Markov chain transition probabilities are governed by the Metropolis-Hastings criterion [24,25].

Multiple Markov chains are run in parallel and then Metropolis-coupled, a method in which chains attempt to swap their current states [26]. Metropolis-coupled chains are known to improve the mixing of parameter values [27] and also convergence behavior [28]. Metropolis-coupled chain *x *can be assigned a heating term (*β*_*x*_) to modify the Metropolis-Hastings transition probability from the current state ***λ ***to a proposed state ***λ'***. This modified transition probability (*U*) is given by the equation(9)

where *q*(***λ ***→ ***λ'***) is the probability of proposing a move from state ***λ ***to ***λ'***. The probability that an attempted swap of parameter values between two randomly selected chains *x *and *y *is successful can be written as(10)

[27]. For the purposes of the present study, an incremental heating scheme was used for eight Metropolis-coupled chains; in this scheme, the heating term for chain *x *is given by β_*x *_= 1/[1 + Δ*K*(*x *- 1)]. A temperature increment parameter of Δ*K *= 1.1 is used in this study, which yields an average swapping rate of 30-40% between chains. Only the state of the non-heated chain is recorded at each step.

The general Metropolis-coupled Markov chain Monte Carlo (MCMCMC) algorithm proceeds as follows:

1) Randomly assign initial parameters value in ***λ***, sampled from *f *(***λ***).

2) Sample *r *genealogies, each with probability Pr (_*r*_*|****λ***), and calculate *f *(***λ****| ***S**) from equation (8).

3) Randomly select a parameter in ***λ ***and propose a new value from *f *(***λ***) to obtain ***λ'***.

4) With probability *U*, let ***λ ***= ***λ'***, otherwise retain ***λ***.

5) Randomly select two chains with states ***λ***_*x *_and ***λ***_*y *_and, with probability *V*, exchange the values of ***λ***_*x *_and ***λ***_*y*_, otherwise retain the current state of each chain.

6) Go to step 2.

In step 6, the algorithm returns to step 2, rather than step 3. This means that at each step the likelihood of the current state is recalculated, rather than retained from the previous step. Retaining the previous likelihood may result in the chain becoming stuck in a state that, by chance from the Monte Carlo sample, yields an unusually high likelihood; however it is also guaranteed to produce samples from the true posterior distribution, regardless of the Monte Carlo sample size [29]. While the true variance of the target distribution can be obtained with this "sticky" method, it may also impede the convergence behavior of the chain and, hence, require more steps in the chain. In contrast, the practice of recalculating the likelihood ("smooth" MCMC) are expected to result in higher acceptance rates, while the resulting posterior distribution may also have increased variance over that of the true target distribution, but it may also improve convergence behavior [30]. Initial runs using the "sticky" MCMC approach yielded lower overall rates of parameter mixing than did the "smooth" MCMC method. Therefore, only results using the "smooth" MCMC algorithm are reported here.

For each dataset, ten independently seeded replicates of the Markov chain are run for 10^5 ^steps, not including an initial "burn-in" period of 10^3 ^steps. A C++ program, written to perform the MCMCMC method called mc3 is freely available over the internet at http://www.rochester.edu/College/BIO/labs/Garrigan/software.htm. The OpenMP application program interface http://www.openmp.org/ is used to distribute parallelized Markov chains across the shared memory of eight cores in dual Intel Quad-core Xeon processors running at 2.66 GHz. The duration of the Metropolis-coupled runs are typically 40-80 hours, depending upon the dataset or the MCMC algorithm. The mc3 program allows users to input the desired number of Metropolis-coupled chains to run on their particular computer.

The potential scale reduction factor (PSRF) is used to quantify the convergence of the ten replicate runs to the stationary distribution [31], as implemented in the CODA package for the free statistical programming environment Rhttp://www.r-project.org. The CODA package is also used to calculate posterior probability densities, parameter autocorrelations and cross-correlations, as well as the 95% highest posterior density (HPD) intervals.

### Assessing the Accuracy of the Method

Twelve simulated joint frequency spectra are generated under a two-population divergence model with known parameters, given in Table 1. Marginal posterior probability distributions for the model parameters are then estimated, using the method outlined above. For each simulated data set, a joint frequency spectrum of 1000 unlinked single nucleotide polymorphism loci is generated for both the X chromosome and the autosomes, resulting in a total of 2000 unlinked polymorphic sites. The number of sampled chromosomes in each simulation is *n*_1 _= 20 and *n*_2 _= 20, for both the X-linked and autosomal joint frequency spectra. The prior distributions used for the analysis of simulated data sets A-F are the same as those given above for the analysis of the human resequence data, except the prior for *α*_1 _was exponentially distributed with mean 50. For simulated data sets G-L, only the prior for *t*_1 _was altered to a uniform distribution over the interval (0, 5).

### Application to Human Resequence Data

The composite likelihood method is then applied to the X-linked and autosomal resequence data of Wall et al. [14]. The data consist of 14 X chromosomes and 28 autosomes sampled from the Mandenka population, a sub-Saharan Africa food-producing population, 16 X chromosomes and 32 autosomes sampled from the Han Chinese population, 16 X chromosomes and 32 autosomes sampled from the Basque population from France, and 14 X chromosomes and 18 autosomes sampled from the Melanesian population. For each population, 20 autosomal and 20 X-linked loci are resequenced for a total of ~210 kilobases from each individual. Using these four datasets, three pairwise analyses are performed: (1) Mandenka-Han Chinese for the Africa-Asia (AA) comparison with 318 X-linked polymorphisms and 655 autosomal polymorphisms, (2) Mandenka-Basque for the Africa-Europe (AE) comparison with a total of 328 X-linked polymorphisms and 648 autosomal polymorphisms, and (3) Mandenka-Melanesia for the Africa-Oceania (AO) comparison with 148 X-linked polymorphisms and 614 autosomal polymorphisms.

One interesting question is whether the parameters of a putative non-African bottleneck or divergence times are consistent across all three comparisons, suggesting a common historical event shared by all non-African populations, or if there is clear evidence for distinct, independent historical divergence/bottleneck events for different non-African populations. These resequence data were chosen because they likely represent neutrally evolving regions of the genome and do not suffer from the ascertainment bias that may artificially skew the frequency spectra of other single nucleotide polymorphism datasets [14].

Another motivation to utilize the resequence data of Wall et al. [14] is that Hammer et al. [21] find that, after correcting for mutation rate, nucleotide diversity for the X-linked loci is nearly equal to the nucleotide diversity for the autosomal loci. Hammer et al. [21] conclude that high variance in male reproductive success may account for the nearly equal effective population sizes of the X and autosomes; however, Pool and Nielsen [20] show that the X *versus *autosomal effective size may be equal during the growth phase following a population bottleneck. The intention here is to use the method to ascertain whether rapid population growth following a bottleneck, or a decrease in male effective population size, may result in increased levels of X chromosome genetic diversity.

## Authors' contributions

D.G. designed the study, wrote the MC3 program, performed the analyses and wrote the paper.
